# A case report: combined posterior subthalamic area and globus pallidus internus deep brain stimulation in Parkinson’s disease

**DOI:** 10.3389/fnhum.2025.1638834

**Published:** 2025-07-29

**Authors:** Qi Deng, Yanghong Zou, Yingwang Yuan, Xin Geng

**Affiliations:** ^1^The Second Department of Neurosurgery, The First Affiliated Hospital of Kunming Medical University, Kunming, Yunnan, China; ^2^NHC Key Lab of Drug Addiction Medicine, Kunming Medical University, Kunming, Yunnan, China

**Keywords:** different targets, deep brain stimulation, posterior subthalamic area, globus pallidus internus, Parkinson’s disease

## Abstract

**Background:**

Deep brain stimulation is a primary surgical treatment for advanced Parkinson’s disease (PD). The globus pallidus interna (GPi) is a key target for this procedure. The posterior subthalamic area (PSA) serves as an effective target for tremor-dominant Parkinson’s disease. However, it is less commonly utilized in conventional DBS surgery compared to the subthalamic nucleus (STN) or the ventral intermediate nucleus (VIM). There is currently no clinical research on the combined DBS surgery targeting both the PSA and the GPi, which is why we have conducted this study.

**Case report:**

We introduced a case of a patient with advanced PD. Due to the patient’s primary manifestations of right-sided tremor and left-sided rigidity, along with significant dyskinesia on the left side, DBS implantation was performed in the left hemisphere targeting the PSA and in the right hemisphere targeting the GPi. The patient’s UPDRS-III score decreased from 73 to 46 postoperatively, showing an improvement of approximately 36.99%, while the H-Y stage improved from stage 4 to 2.5, representing a 37.5% improvement. During the 6-months postoperative follow-up, the patient’s PD symptoms were effectively controlled, with no significant adverse effects.

**Discussion:**

When advanced PD patients present with asymmetric and variable motor symptoms, combined DBS stimulation targeting both the GPi and the PSA is a viable treatment option.

## 1 Introduction

Parkinson’s disease is the second most common neurodegenerative disorder after Alzheimer’s disease, and its onset is generally believed to be associated with the depletion of dopamine in the nigrostriatal pathway (Dauer and Przedborski, 2003). The disease leads to impairments in both motor and non-motor functions, and its high cost of care and treatment significantly increases the economic burden on families and society in the context of an aging population. Deep brain stimulation (DBS) is the primary surgical treatment for primary PD, and it can improve specific symptoms by targeting different brain regions (Abusrair et al., 2022). Currently, most PD patients exhibit asymmetrical symptoms on the left and right sides. If we use bilateral symmetric target DBS surgery, it may not effectively address the issue of asymmetrical symptoms. The globus pallidus interna (GPi) is a target that has a direct antiparkinsonian effect, particularly in reducing dystonia (Vidailhet et al., 2005). The posterior subthalamic area (PSA) is a novel target that demonstrates better efficacy in alleviating tremors (Ramirez-Zamora et al., 2016).

Here, we present a case of a PD patient who primarily presented with right-sided tremor, left-sided rigidity, and significant dyskinesia on the left side. Given the patient’s severe right-sided tremor and the superior efficacy of the PSA over both the subthalamic nucleus (STN) and GPi for tremor control, we selected the PSA as the target in the left cerebral hemisphere, while opting for GPi stimulation in the right hemisphere to address concurrent non-motor symptoms and left-sided dyskinesia.

The use of asymmetric target stimulation during surgery has gradually been adopted in clinical practice and has received positive feedback (Schadt et al., 2007; Hedera et al., 2013; Maesawa et al., 2022). However, there is still no consensus on the optimal target for DBS, and research on asymmetric targets targeting the PSA has primarily focused on essential tremor, with most studies employing a combination of the PSA and the VIM (ventral intermediate nucleus) stimulation (Yilmaz et al., 2024; Chong et al., 2024; Kojoh et al., 2020). Research on the asymmetric targeting of the GPi has only been reported in cases where GPi and subthalamic nucleus (STN) stimulation were combined to treat PD, primarily characterized by tremor (Zeng et al., 2022). Zhang et al. (2020) demonstrated the efficacy of combined STN-GPi DBS in Parkinson’s disease through a study involving eight patients, particularly for those with poor contralateral symptom control or requiring medication reduction. In contrast to these existing asymmetric DBS approaches, we present the first reported PSA-GPi combination for PD patients exhibiting rigidity-dyskinesia asymmetry.

The combined use of the GPi and the PSA in DBS surgery remains to be further explored. Although numerous reports have emerged regarding the use of different targets in DBS surgery, to our knowledge, there are scant clinical cases involving the combined treatment of GPi-PSA. Today, we will present the technical approach and therapeutic outcomes of this novel treatment strategy.

## 2 Case presentation

### 2.1 Presentation and examination

This case report describes a 71-years-old male patient who developed right upper limb tremor without identifiable triggers 5 years ago, with subsequent progressive spread to the right lower limb, left upper limb, and ultimately the left lower limb, resulting in generalized tremor involving all four extremities. He was diagnosed with “Parkinson’s disease” at a local hospital. After treatment with half a tablet of carbidopa-levodopa (1/2 tablet daily), his condition was well-controlled. Over the past 2 years, his symptoms have progressively worsened, manifesting as prominent right-sided limb tremor (6 Hz frequency), left-sided rigidity with tremor (3 Hz frequency), bradykinesia, turning difficulty, impaired nocturnal turning, and dysphagia. The patient is currently being treated with half a tablet of carbidopa-levodopa (1/2 tablet) four times a day (qid), 1 tablet of amantadine once a night (qn), and 1 tablet of pramipexole three times a day (tid). However, the symptoms have not improved significantly, and the patient has developed dyskinesias and other adverse effects from the medication. As a result, the patient has sought further treatment at this hospital for surgical intervention. Physical examination showed that the patient’s facial expression was stiff, the neck muscle tension was high, the limb muscle strength was grade 5, the right limb muscle tension was increased, the tremor was obvious, the left limb muscle tension was increased, the knee joint was stiff, the joint activity was slow, and the stability was poor. In the upright state, the back leans forward, the walking is unstable, and the turning is slow. Bilateral finger-nose test was positive, UPDRS-III was 73, H-Y stage was 4, PDQ-39 score was 118, ALCT showed a 57 % improvement rate, and DBS surgery was recommended. The patient did not report cardiovascular, pulmonary, renal, or endocrine diseases. There was no abnormality in the MRI images of the patient’s brain.

### 2.2 Surgical interventions

Given the patient’s severe right-sided tremor, we selected the left PSA as the target for stimulation. Considering the left-sided rigidity and prominent dyskinesia, we chose the right GPi as the second target. Therefore, we performed bilateral DBS with a dual lead configuration, targeting the left PSA and the right GPi. The patient’s CT images were acquired using the Leksell Stereotactic Frame System and fused with preoperative MRI images. The surgical plan was created using the Leksell Stereotactic Frame System. Local anesthesia was administered first, followed by the fixation of the patient using the Leksell stereotactic frame.

**FIGURE 1:**
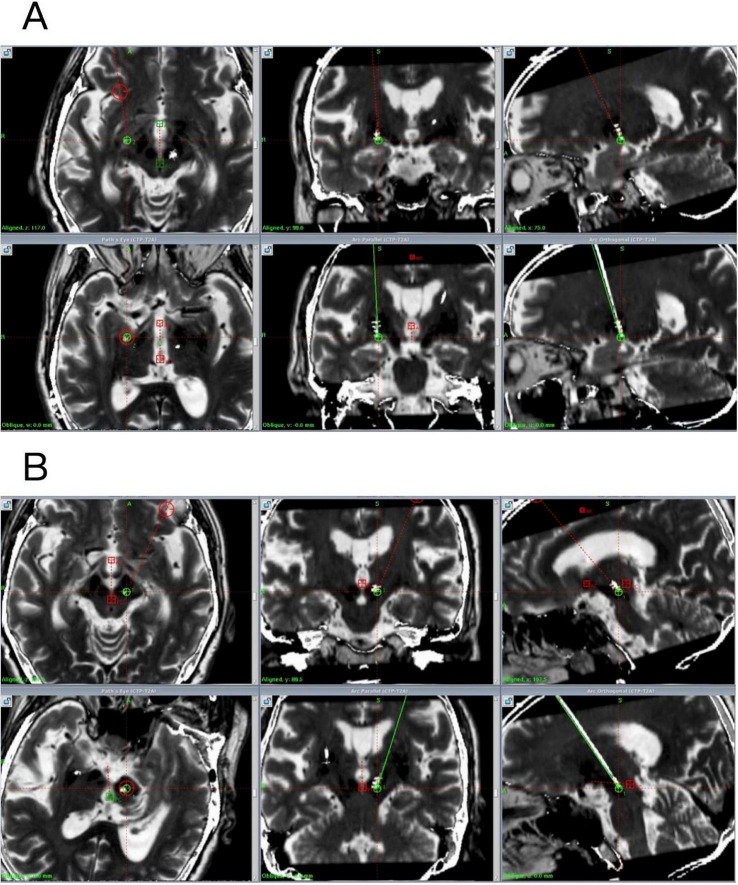
Preoperative and postoperative fused images (preoperative plan in green, actual implanted electrode shown in white). **(A)** Postoperative fused image of the right GPi. **(B)** Postoperative fused image of the left PSA.

The X coordinate of the left PSA target is located at 107.5 mm on the left side of the midpoint of the AC-PC line, the Y coordinate is located at 89.5 mm behind the midpoint of the AC-PC line, and the Z coordinate is located at 121 mm below the midpoint of the AC-PC line. The angle between PSA target and AC-PC plane was 107.5°, and the angle between PSA target and midline was 57°. The X coordinate of the right GPi target is located on the right side of the midpoint of the AC-PC line at 75 mm, the Y coordinate is located 99 mm in front of the midpoint of the AC-PC line, and the Z coordinate is located 117 mm below the midpoint of the AC-PC line. The angle between GPi target and AC-PC plane was 87°, and the angle between GPi target and midline was 73° (Figure 1).

The patient underwent intraoperative electrophysiological mapping using the Alpha Omega microelectrode recording system to assess the functional areas of the target nuclei. The microelectrode recorded signals from the left PSA and the right GPi. During the procedure, experimental stimulation was performed, and the patient showed significant improvement in tremor symptoms without any adverse effects. After thoroughly disinfecting the right occipital, posterior auricular, and cervical regions, as well as the right subclavicular area, the patient was administered local anesthesia. The pulse generator (G102RZ) was implanted, and the lead was finally placed. The procedure was completed successfully, with no complications during or after surgery. The patient was conscious and stable at the end of the operation.

### 2.3 Postoperative course

The patient exhibited a good mental state postoperatively, with a significant reduction in tremor and the ability to perform daily activities independently, including ambulation without assistance. Postoperative image fusion confirmed precise electrode positioning without intracranial complications, utilizing T1-weighted contrast-enhanced (1 mm slice thickness), standard T1-weighted, axial and coronal T2-weighted, and susceptibility-weighted imaging (SWI, 2 mm slice thickness) sequences, with fusion processing performed using Leksell swgiplem software (version 10.0) (Figure 1). Consistent with both the product requirements for bilateral uniform frequency settings and the clinically conventional 130 Hz stimulation paradigm, initial stimulator parameters were configured at: amplitude 1.2 V, pulse width 60 μs (microseconds), and frequency 130 Hz upon device activation.

When stimulating the left PSA, the patient’s right-sided tremor was significantly reduced, and when stimulating the right GPi, the patient’s left-sided rigidity improved markedly. The patient’s postoperative ON-DBS UPDRS-III score of 46 demonstrated a 36.99% improvement compared to the preoperative OFF-DBS score of 73, with tremor frequencies bilaterally improved from preoperative levels (right: 6 Hz; left: 3 Hz) to 2 Hz in both extremities, while the Hoehn and Yahr stage improved from 4 to 2.5 postoperatively, representing a 37.5% enhancement in disease severity (Table 1). The patient continued with the same medication regimen for PD and underwent regular follow-up. At the 6-months postoperative follow-up, the patient was re-evaluated, and a DBS programming session was conducted. The patient’s Parkinson’s disease symptoms were effectively controlled postoperatively, with a MoCA score of 27 indicating preserved cognitive function and no significant adverse effects observed during the initial follow-up period; scheduled longitudinal follow-ups will be conducted to monitor the sustained therapeutic efficacy of this surgical intervention.

**TABLE 1:** Comparison of pre- and postoperative scale scores in the patient.

Status	UPDRS-III	H-Y stage
Preoperative	73	4
Postoperative	46	2.5
Improvement	36.99%	37.50%

## 3 Discussion

Currently, four primary targets are utilized in DBS surgery: the ventral intermediate nucleus (VIM), STN, GPi, and PSA. The VIM, located within the ventrolateral thalamus, demonstrates superior efficacy for tremor control and is indicated for essential tremor, Parkinson’s disease with isolated tremor symptoms, and tremor-dominant Parkinson’s disease subtypes (Mao et al., 2019). However, current clinical trials demonstrate that although this target shows satisfactory short-term therapeutic efficacy, it exhibits poor long-term tolerability with progressively diminishing treatment effects over time (Blomstedt et al., 2007). Moreover, this target is associated with significant adverse effects, including dysphagia, gait disturbances, and postural instability (Hariz and Blomstedt, 2022). Consequently, considering the long-term quality of life outcomes, we did not prioritize this target as the primary therapeutic option in the current treatment regimen.

The STN, located within the basal ganglia, remains a classical and pivotal target for DBS surgery, with extensive clinical evidence demonstrating its efficacy in alleviating tremor, rigidity, and bradykinesia in the majority of PD patients (Kocabicak et al., 2012). The GPi, an integral component of the basal ganglia (BG) and a classical DBS target, is anatomically composed of the medial (GPi) and lateral (GPe) segments. The GPi-DBS restores the balance of the basal ganglia circuitry by inhibiting the hyperactive neurons in the GPi and simultaneously suppresses pathological β-band oscillations while enhancing γ-band oscillations to improve motor control. Randomized controlled trials have shown that GPi-DBS improves baseline UPDRS motor scores during the off-medication state by 27%–54% (Au et al., 2021). Currently, while some researchers contend that the STN demonstrates superior efficacy over the GPi for tremor amelioration, others propose that the GPi may constitute the tremorgenic source, resulting in ongoing controversy regarding optimal target selection (STN versus GPi) for tremor management in clinical practice (Wong et al., 2020; Dirkx et al., 2016). However, a consensus exists regarding the suboptimal tremor control efficacy of both the STN and GPi, particularly in patients with high-frequency tremor manifestations (Azghadi et al., 2022; Wong et al., 2020). In this case, the patient exhibited high-frequency right-sided tremor (6 Hz) with potential comorbid essential tremor components, for which both conventional targets demonstrated limited therapeutic efficacy, prompting our exploratory investigation of the PSA as an alternative intervention target.

The PSA is located posterior to the ventral thalamus and is primarily composed of the caudal zona incerta (cZi), the dentato-rubro-thalamic tract (DRTT), and adjacent fibers. Mathematical theory model simulations suggest that PSA-DBS may reduce the abnormal signals transmitted from the cerebellum to the thalamus by inhibiting the pathological β-oscillations in the DRTT (Wu et al., 2023). Clinical trials have demonstrated that the PSA exhibits markedly superior efficacy in tremor control compared to alternative DBS targets (Kim et al., 2021). Postoperative outcomes of the PSA-DBS are significantly improved: patients typically experience an improvement of 80%–95% in symptoms after medication discontinuation, and long-term follow-up studies show that tremor improvement can last for more than 5 years (Stenmark Persson et al., 2023); (Chopra et al., 2013). Moreover, this target is primarily associated with dysphagia and balance disorders as its main adverse effects, while demonstrating a significantly lower incidence of complications compared to other nuclear targets (Xie et al., 2012; Chopra et al., 2013). Based on its superior efficacy in controlling both tremor and gait disturbances compared to STN and GPi, coupled with a more favorable adverse effect profile, we selected this target in the left cerebral hemisphere to manage the patient’s severe right-sided limb tremor symptoms.

The patient exhibited left-sided limb rigidity accompanied by dyskinetic movements, a clinical presentation for which the GPi is typically preferred over the STN in standard therapeutic practice (Sriram et al., 2014; Mirza et al., 2017). The patient presented with a comprehensive symptom profile encompassing motor manifestations (tremor, rigidity, bradykinesia, and gait disturbances) alongside non-motor features including depression, anxiety, cognitive impairment, and constipation, for which the GPi target demonstrates superior therapeutic efficacy over the STN in addressing cognitive dysfunction, anxiety, and depressive symptoms (Wang et al., 2016; El Ghazal et al., 2023). Given the GPi’s demonstrated superiority in managing both dyskinesia and non-motor symptoms, we ultimately selected the GPi target for implantation in the patient’s right cerebral hemisphere rather than the STN.

In recent years, with the discovery of different target areas, asymmetric target surgery has attracted the attention of researchers. Zhang et al. (2020) recently demonstrated the therapeutic efficacy of combined STN-GPi DBS surgery in eight Parkinson’s disease patients through comparative analysis of UPDRS-III scores, Timed Up and Go (TUG) test results, PDQ-39 questionnaire outcomes, and axial symptom assessments performed preoperatively, immediately postoperatively, and at 6- and 12-months follow-ups. In previous reports, the combination of the GPi and the STN is more effective in improving symptoms on the contralateral limb than the GPi or the STN alone (Zeng et al., 2022). The PSA-DBS has been used in multiple clinical cases for combining the PSA-VIM stimulation to treat tremor syndromes or essential tremor (Yilmaz et al., 2024; Chong et al., 2024). Still, it is relatively uncommon in the treatment of PD tremor, with only one case report demonstrating a successful outcome of combining the PSA-VIM stimulation for Parkinsonian tremor symptoms (Kojoh et al., 2020). There have been no clinical reports of DBS surgery targeting the combined PSA-GPi pathway.

The current DBS systems can use a single pulse generator device to adjust stimulation for two electrode leads. Therefore, in patients with PD primarily characterized by tremor, using multiple-target approaches with dual-electrode configurations is technically feasible and more effective. In this case, a severe tremor in PD may involve complex pathophysiology affecting multiple functional networks, including the cerebellum-thalamocortical pathway and the globus pallidus-thalamocortical pathway.

The combined treatment of the left PSA and the right GPi can simultaneously target both the tremor circuit and the overall motor control network. This combined approach demonstrated superior efficacy over other target combinations in managing the patient’s overall non-motor symptoms, high-frequency right-sided limb tremor, and left-sided rigidity with dyskinetic movements, while maintaining a relatively favorable adverse effect profile.

It is important to note several limitations. First, the stimulation frequencies for the PSA and the GPi differ, requiring separate frequency adjustments during DBS programming. In the absence of prior reference cases and due to product requirements mandating identical bilateral frequency settings, we proceeded with the intervention based on established clinical experience. We recommend that future clinical trials gradually determine the optimal frequency settings for the PSA-GPi combination (Hidding et al., 2023). As this study constitutes a single-case report with only 6-months follow-up data, the observed outcomes may reflect incidental findings; we plan to conduct extended longitudinal monitoring to verify the surgical efficacy, while definitive confirmation of PSA-GPi DBS’s therapeutic effects for Parkinson’s disease patients with asymmetric bilateral motor symptoms will require future randomized controlled trials for validation.

## 4 Conclusion

We report the first documented case utilizing combined PSA-GPi DBS to treat Parkinson’s disease presenting with unilateral tremor and rigidity accompanied by asymmetric motor symptoms, with our findings suggesting potential efficacy in managing rigidity-dyskinesia asymmetry; however, as this represents a single-case study, future randomized controlled trials are warranted to definitively establish the therapeutic value of this intervention. However, the long-term effects of dual-target DBS stimulation at the PSA-GPi interface remain unclear and require further investigation and long-term monitoring to fully assess its safety and efficacy.

## Data Availability

The original contributions presented in this study are included in this article/supplementary material, further inquiries can be directed to the corresponding author.
